# Self‐Assembly Behavior of Monodisperse PEG Amphiphiles Bearing Hydrophobic Units with Distinct Molecular Shapes in Water

**DOI:** 10.1002/open.70214

**Published:** 2026-04-20

**Authors:** Ai Kohata, Rei Hamaguchi, Kazushi Kinbara

**Affiliations:** ^1^ School of Life Science and Technology Institute of Science Tokyo Yokohama‐shi Kanagawa Japan; ^2^ Research Center for Autonomous Systems Materialogy (ASMat) Institute of Integrated Research (IIR) Institute of Science Tokyo Yokohama‐shi Kanagawa Japan

**Keywords:** amphiphile, PEG, self‐assembly, shape, supramolecular chemistry, surfactant

## Abstract

The shape of hydrophobic units on the self‐assembly of amphiphiles in pure water remains underexplored. In this work, three types of octaethylene glycol (OEG)–appended amphiphiles bearing propeller‐shaped, twisted, and planar hydrophobic units were synthesized. They formed different types of assemblies, such as liquid droplets, nanosheets, and supramolecular oligomers, suggesting the importance of molecular shape in designing the self‐assembly of amphiphiles in water.

## Graphical abstract

Three types of PEG amphiphiles bearing hydrophobic units with distinct molecular shapes, such as twisted *p*‐terphenyl, propeller‐shaped trityl, and planar pyrenyl groups, self‐assembled in pure water to form nanosheets, liquid droplets, and supramolecular oligomers, respectively. By using PEG with a defined molecular weight as a hydrophilic unit, we investigated how the shape of hydrophobic units affects the assembly of nonionic amphiphiles.

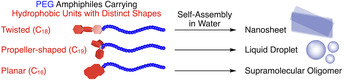

## 1 Introduction

Self‐assembly of amphiphiles in aqueous media has long been an active area of research in materials science and biomedicine [1, 2]. A wide variety of assembled objects, such as particles, droplets, tubes, or sheets ranging from nano‐ to micro‐scales, can be formed in water and exhibit distinctive intrinsic properties [3, 4]. For designing such assemblies, the shape of the hydrophobic unit is crucial [5, 6, 7, 8], because the hydrophobic effect dominates the assembly process in water [9, 10]. Although the formation of assembled structures can be computationally predicted [11] by using a concept of the critical packing parameters [12, 13], such methodologies often underestimate the role of hydrophobic parts [14] and directional intermolecular interactions in the assembly [15]. To date, the planar hydrophobic units were frequently employed together with functional groups that can induce directional forces such as hydrogen‐bonding and salt‐bridge interactions [16, 17, 18, 19], whereas nonplanar units were rarely explored because their nonplanar configurations spatially hinder the intermolecular orientation and stacking, often resulting in the formation of disordered aggregates. In addition, the previously reported structures were often prepared in a mixture of organic solvents and water [20, 21, 22, 23, 24] because monomers cannot be fully dissolved in pure water. Therefore, the self‐assembled materials using nonplanar units formed in pure water, without organic cosolvents, remain underexplored [6]. In this work, we report the assembling behavior of octaethylene glycol (OEG)–appended nonionic amphiphiles bearing hydrophobic units with three different shapes in pure water.

**FIGURE 1:**
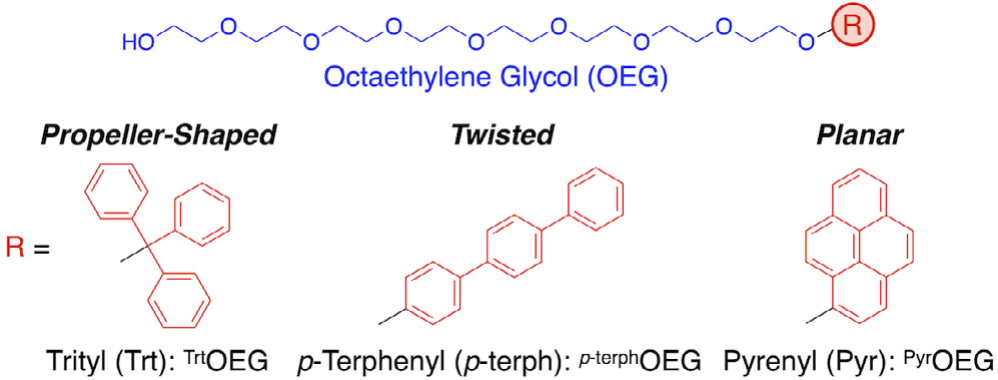
Molecular structures of octaethylene glycol (OEG) amphiphiles bearing a propeller‐shaped trityl (Trt) group (^Trt^OEG), a twisted *p*‐terphenyl (*p*‐terph) group (^*p*‐terph^OEG), and a planar pyrenyl (Pyr) group (^Pyr^OEG).

Poly(ethylene glycol) (PEG) is a widely used hydrophilic unit in nonionic monomers owing to its high water solubility [25]. We recently reported that the water solubility of PEG‐appended molecules increases with long ethylene glycol chains, using both experimental and computational approaches [26]. Because the water solubility governs the boundaries of dissolution, phase separation, and self‐assembly, the use of PEGs with defined lengths [27, 28, 29] can provide a structurally consistent framework for comparison, which would allow us to reveal the impact of the shape of hydrophobic units on their self‐assembly.We synthesized three types of OEG‐appended amphiphiles bearing hydrophobic units with similar numbers of carbon atoms with different shapes, including propeller‐shaped trityl (Trt, C_19_; ^Trt^OEG), twisted *p*‐terphenyl (*p*‐terph, C_18_; ^*p*‐terph^OEG), and planar pyrenyl (Pyr, C_16_; ^Pyr^OEG) groups (Figure 1). Under identical concentration and temperature, ^Trt^OEG, ^*p*‐terph^OEG, and ^Pyr^OEG formed liquid droplets, nanosheets, and supramolecular oligomers, respectively.

## 2 Results and Discussion

A series of OEG amphiphiles was synthesized according to a method analogous to the reported procedures [26, 27]. In brief, OEG was reacted with 4‐hydroxy‐*p*‐terphenyl and 1‐hydroxypyrene, via Williamson ether synthesis, affording ^*p*‐terph^OEG and ^Pyr^OEG, respectively. A substitution reaction between trityl chloride and OEG yielded ^Trt^OEG. Three OEG amphiphiles thus obtained were diluted with ultrapure water at room temperature. First, to determine the concentration ranges at which the OEG amphiphiles start to aggregate, their critical aggregation concentration (CAC) was evaluated from the surface tension measurements (Figure S1). The CAC of ^Pyr^OEG ([^Pyr^OEG]_CAC_ = 148 µM) was smaller than that of ^Trt^OEG ([^Trt^OEG]_CAC_ = 361 µM) despite having fewer carbon atoms in the hydrophobic unit (C_16_) than ^Trt^OEG. In addition, the surface tension of the aqueous solution of ^Pyr^OEG at 10 mM (51.7 ± 0.41mN/m) was higher than that of ^Trt^OEG (45.5 ± 0.08 mN/m). These results indicate that ^Pyr^OEG with a planar Pyr group can interact strongly with one another, possibly via π–π interactions. Notedly, the CAC of ^*p*‐terph^OEG could not be determined due to its poor water solubility (<1 µM) as previously reported [26]. This clear difference in CAC among ^Trt^OEG, ^*p*‐terph^OEG, and ^Pyr^OEG can be highly attributed to their shape rather than the number of carbon atoms.

Next, the water solubility of three OEG amphiphiles was investigated by measuring the optical density (OD) of their bulk solutions (*λ* = 560 nm). The aqueous solution of ^Trt^OEG became turbid around 0.5 mM (Figure S2), while that of ^Pyr^OEG remained transparent up to 1.7 M [26]. Since the fluorescent signal at 504 nm, originating from the pyrene excimer, emerged above the CAC of ^Pyr^OEG (Figure S3), the formation of a pyrene dimer was plausible. However, the dimeric or oligomeric assemblies of ^Pyr^OEG did not grow into sufficiently large assemblies to make the solution turbid. When the supernatant of the ^*p*‐terph^OEG suspension was analyzed by the absorption spectrometer, its concentration was found to be below the detection limit (<1 µM) [26].

**FIGURE 2:**
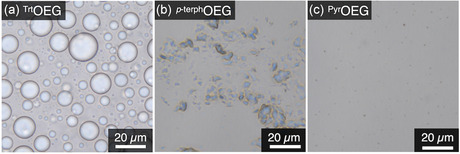
Optical micrographs of aqueous solutions of (a) ^Trt^OEG, (b) ^*p*‐terph^OEG, and (c) ^Pyr^OEG ([^Trt^OEG] = [^*p*‐terph^OEG] = [^Pyr^OEG] = 10 mM) at 25°C.

**FIGURE 3:**
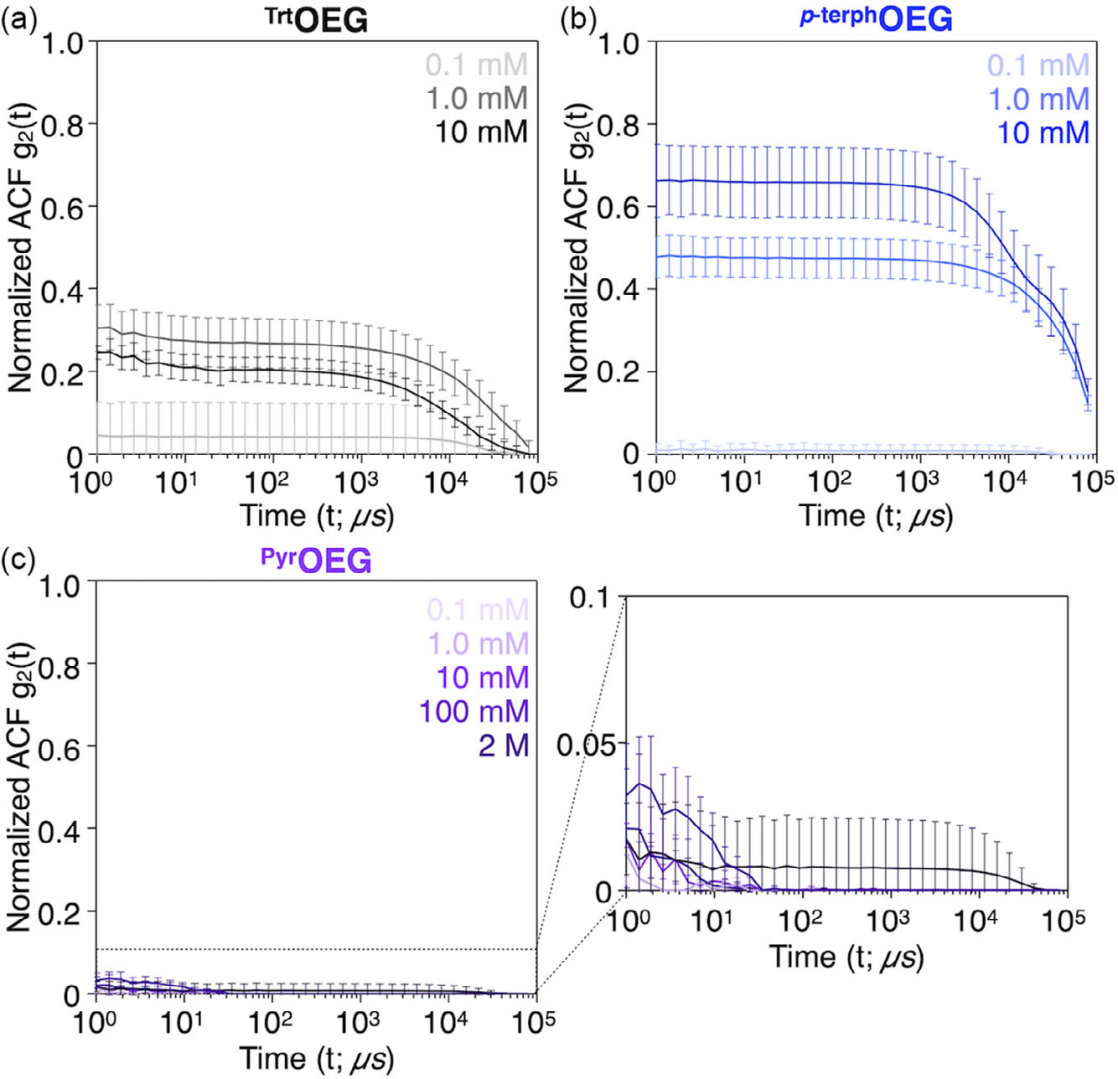
Normalized autocorrelation functions (ACFs) of (a) ^Trt^OEG, (b) ^*p*‐terph^OEG ([^Trt^OEG] = [^*p*‐terph^OEG] = 0.1, 1.0, 10 mM), and (c) ^Pyr^OEG ([^Pyr^OEG] = 0.1, 1.0, 10, 100 mM, 2 M) and its magnified graphs in water at 20°C. Error bars represent the standard deviations of three independent experiments (*n* = 3).

**FIGURE 4:**
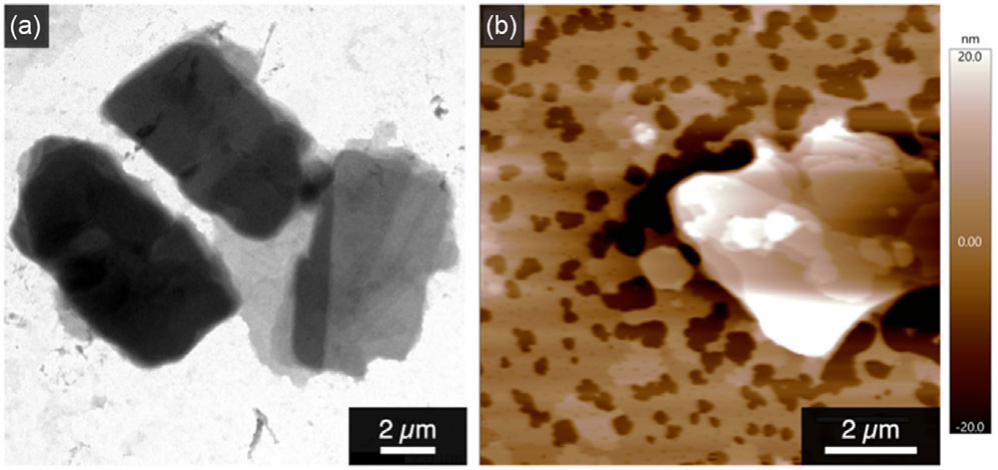
(a) Transmission electron micrograph and (b) atomic force micrograph of ^*p*‐terph^OEG assemblies prepared from its aqueous suspension at 0.1 mM.

Finally, we studied whether OEG amphiphiles form into any assemblies at the microscale. When a portion of the turbid ^Trt^OEG suspension ([^Trt^OEG] = 10 mM) was cast onto a glass substrate, interestingly, we observed micrometer‐scale spherical objects without internal substructures (Figures 2a and S4a). These microspheres coalesced in water (Supporting Movie S1), indicating they are phase‐separated liquid droplets composed of ^Trt^OEG. On the other hand, in the suspension containing ^*p*‐terph^OEG, a white precipitate was observed (Figure 2b), which was also visualized under a polarized optical microscope (Figure S5), suggesting the formation of highly ordered, crystalline‐like structures in water. Under identical conditions, no mesoscale structure was observed in the aqueous solution of ^Pyr^OEG (Figures 2c and S4b). Accordingly, by dynamic light scattering (DLS), the moderate ACF values were confirmed in the aqueous solution and suspensions of ^Trt^OEG ([^Trt^OEG] = 0.1, 1, 10 mM; Figure 3a), suggesting the formation of polydispersed particles ranging from nanometers to micrometers in size (Figure S6a). Meanwhile, ^*p*‐terph^OEG suspensions ([^*p*‐terph^OEG] = 0.1, 1, 10 mM) exhibited stronger values than those of ^Trt^OEG (Figure 3b), suggesting the presence of large aggregates (Figure S6b). In contrast, the ACF values were considerably low for ^Pyr^OEG even at 2 M (Figure 3c), which were comparable to those of PEG600 without hydrophobic units(Figure S7). These DLS results were consistent with the assemblies observed under optical microscopy and suggest that ^Pyr^OEG plausibly forms oligomeric assemblies. It should be noted that, when the aqueous suspension of ^*p*‐terph^OEG was observed by transmission electron microscope (TEM), sheet‐like structures were visualized (Figure 4a), whose thickness was further revealed to be as thin as 5 nm (Figure S8) by using atomic force microscope (AFM; Figure 4b).

As depicted in Figure 2, ^Trt^OEG, ^*p*‐terph^OEG, and ^Pyr^OEG behaved quite differently in water: the formation of liquid droplets, stacked nanosheets, and assemblies with several molecules, respectively. Taking into account that three OEG amphiphiles carry hydrophobic units with a similar number of carbon atoms, these differences in assemblies should be attributed to their variations in molecular shape. ^*p*‐terph^OEG bearing a twisted *p*‐terphenyl group, in which three benzene rings were connected in one‐dimensional manner with restricted motions, self‐assembled into stacked nanosheets and exhibited poor water solubility (<1 µM). On the other hand, ^Trt^OEG formed phase‐separated liquid droplets with moderate water solubility (0.5 mM), possibly because the freely rotating conformations of a propeller‐shaped Trt group reduce intermolecular π−π interactions and increase the room for hydration of benzene rings. Interestingly, ^Pyr^OEG bearing a planar four‐fused benzene motif self‐assembled into supramolecular oligomers around 150 µM ([^Pyr^OEG]_CAC_), but exhibited remarkably high water solubility (>1.7 M). Even though ^Pyr^OEG was more likely to self‐assemble than ^Trt^OEG ([^Trt^OEG]_CAC_ = 360 µM), ^Pyr^OEG exhibited higher water solubility than ^Trt^OEG, possibly because the pyrene unit formed a stable assembly surrounded by hydrophilic PEG units, which prevented ^Pyr^OEG molecules from forming polymeric assemblies.

## 3 Conclusion

In conclusion, we investigated the self‐assembly of OEG amphiphiles with three different hydrophobic units using a length‐defined PEG chain in pure water. The shapes of hydrophobic units significantly affected not only their water solubility ([^*p*‐terph^OEG] < 0.1 µM, [^Trt^OEG] = 0.5 mM, [^Pyr^OEG] > 1.7 M), but also the self‐assembly behaviors, affording various assemblies such as nanosheets, liquid droplets, and supramolecular oligomers. As demonstrated in this study, the shape of amphiphilic monomers should be considered an important factor in designing and producing functional structures in water, where the intermolecular interactions are dominated by strong hydrophobic effects, complicating the structural prediction of self‐assembly.

## Supporting Information

Additional supporting information can be found online in the Supporting Information section.

## Funding

This work was supported by the Ministry of Education, Culture, Sports, Science, and Technology of Japan (JP23K17363, JP25K18070).

## Conflicts of Interest

The authors declare no conflicts of interest.

## Supporting information

Supplementary Material

## Data Availability

The data that support the findings of this study are available from the corresponding author upon reasonable request.
