# Supplementary material for: Self‐Assembly Behavior of Monodisperse PEG Amphiphiles Bearing Hydrophobic Units with Distinct Molecular Shapes in Water
Source: ChemistryOpen. 2026 Apr 20;15(5):e70214. doi: 10.1002/open.70214 (PMC13096572; doi:10.1002/open.70214)
Supplement: Supplementary file 1 — Supplementary Material [file OPEN-15-e70214-s001.zip › open70214-sup-0002-SuppData-S2.pdf]

Supporting Information

Self-Assembly Behavior of Monodisperse PEG Amphiphiles Bearing Hydrophobic Units with Distinct Molecular Shapes in Water

Ai Kohata,[1] Rei Hamaguchi,[1] Kazushi Kinbara[1,2,*]

[1] *School of Life Science and Technology, Institute of Science Tokyo,*

4259 Nagatsuta-cho, Midori-ku, Yokohama, Kanagawa 226-8501, Japan.

[2] *Research Center for Autonomous Systems Materialogy (ASMat),*

Institute of Integrated Research (IIR), Institute of Science Tokyo,

4259 Nagatsuta-cho, Midori-ku, Yokohama, Kanagawa 226-8501, Japan

Table of Contents

1. **General** **S2**
2. **Surface Tension** **S3**
3. **Optical Density** **S4**
4. **Fluorescent Spectra** **S5**
5. **Transmission Electron Microscopy** **S6**
6. **Polarized Optical Microscopy** **S7**
7. **Dynamic Light Scattering** **S8**
8. **Atomic Force Microscopy** **S9**

1. General

Unless otherwise noted, all commercial reagents were used as received. Surface tension was measured on a KRÜSS model Drop Shape Analyzer DSA100. Electronic absorption spectra were recorded on a JASCO model V-650 spectrophotometer using a quartz cell of 1 mm optical path length. Fluorescent spectra were recorded on a JASCO model FP-8550 spectrofluorometer. Polarized optical microscopy (POM) was performed on an Olympus model BX-53 polarizing optical microscope. Optical microscopy was performed on a Nikon model ECLIPSE Ti2-A. Dynamic light scattering (DLS) measurements were performed using a HORIBA model nanoPartica SZ-100V2 equipped with a 532 nm laser light source. Transmission electron microscopy (TEM) was performed on a JEOL model JEM-1400 electron microscope operating at 100 keV. Atomic force microscopy (AFM) was performed via tapping mode on an Oxford Instruments model Asylum Research Cypher S.

The OEG amphiphiles were evaporated to dryness under reduced pressure and diluted with ultra-pure water to afford their aqueous solutions.

2. Surface Tension

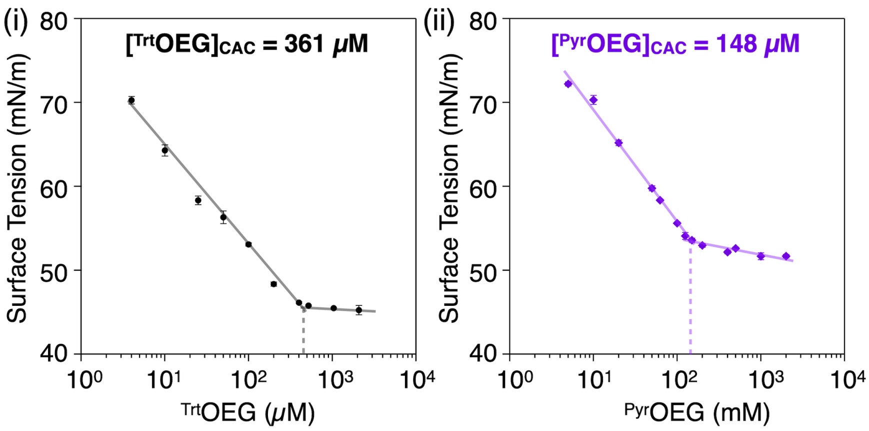

Figure S1. Surface tension vs. concentration plots of aqueous solutions of (i) ^{Trt}OEG and (ii) ^{Pyr}OEG at 20 °C. Their CACs were determined from the intersection of the fitted lines ([^{Trt}OEG]$_{CAC}$ = 361 μM, [^{Pyr}OEG]$_{CAC}$ = 148 μM). Error bars represent the standard deviation (n = 10).

3. Optical Density

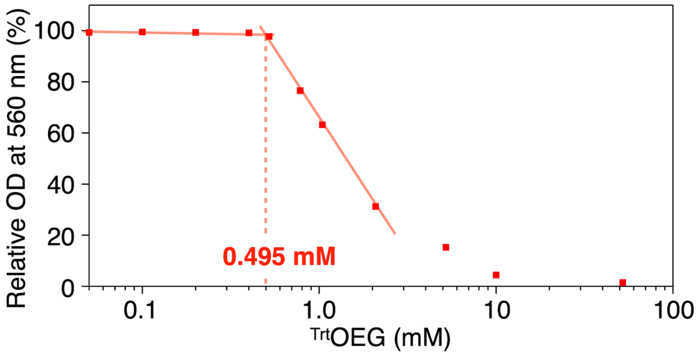

Figure S2. Optical density (OD) changes (λ = 560 nm) of aqueous ^{Trt}OEG (0.05–50 mM) in water at 20 °C.

4. Fluorescence Spectra

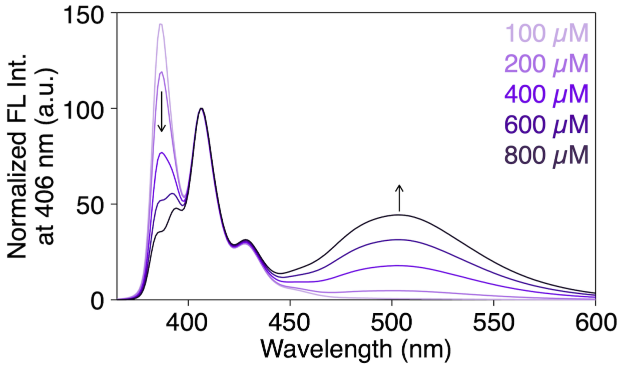

Figure S3. Normalized fluorescence spectra (λ_{ex} = 345 nm) of ^{Pyr}OEG (100–800 μM) in water at 20°C.

5. Transmission Electron Microscope (TEM)

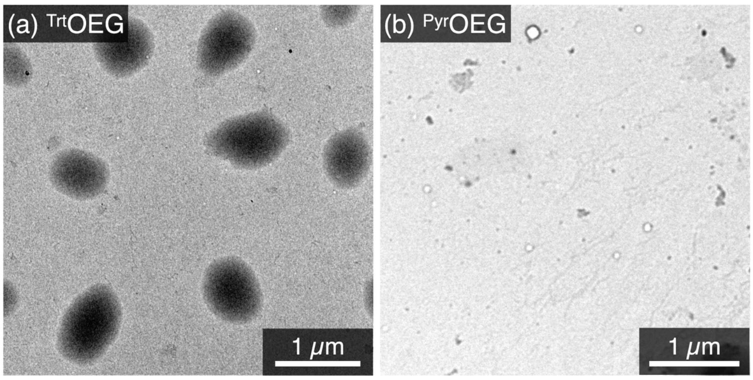

Figure S4. Transmission electron micrographs of (a) ^{Trt}OEG and (b) ^{Pyr}OEG prepared from their aqueous solutions at 5.0 mM.

6. Optical Microscopy

A portion of the solutions of OEG amphiphiles was placed between two pieces of bare glasses with a double-faced adhesive spacer (0.16 mm thick).

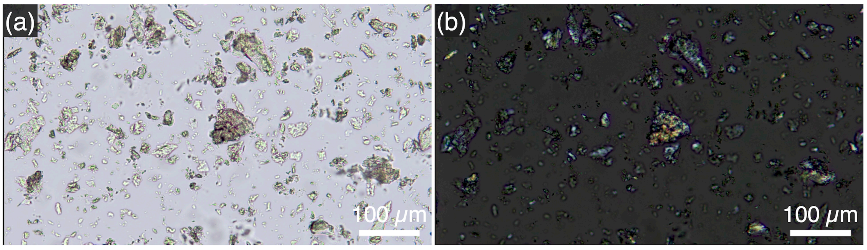

Figure S5. (a) Optical microscope image of $^{p\text{-terph}}$OEG (10 mM) in water at 25 °C and (b) its polarized image under crossed polarizers.

7. Dynamic Light Scattering

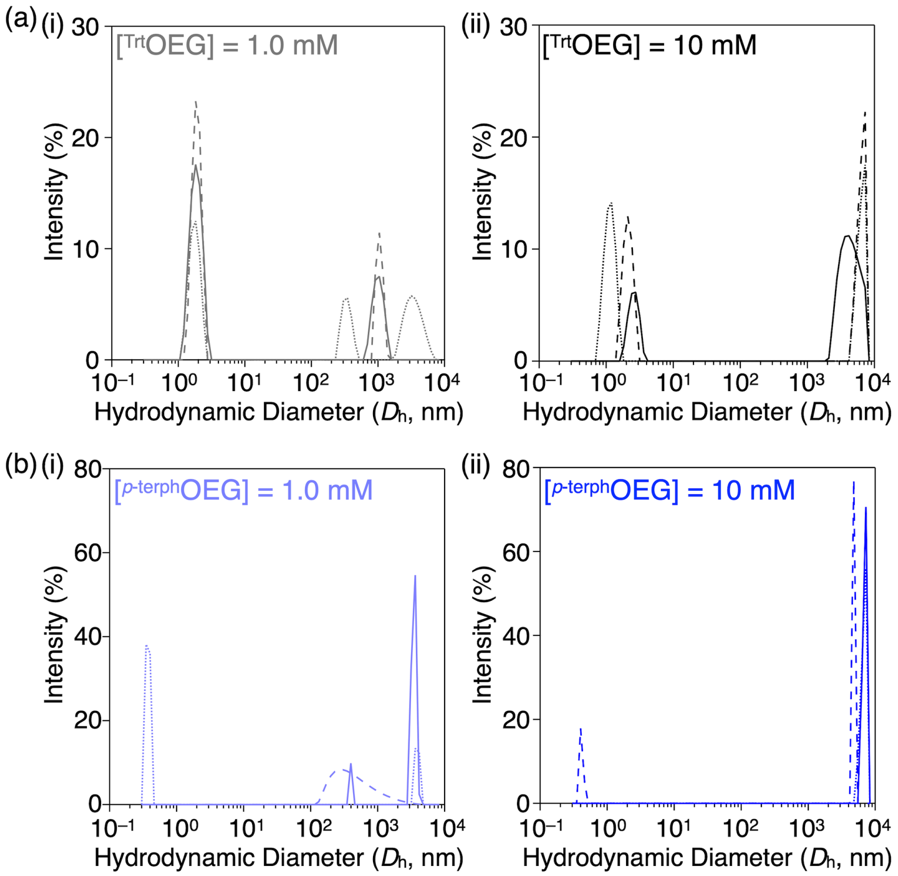

Figure S6. Dynamic light scattering (DLS) profiles (a) ^{Trt}OEG and (b) $^{p\text{-terph}}$OEG at (i) 1.0 mM and (ii) 10 mM in water at 20 °C. Three independent measurements were plotted as solid, dotted, and dashed lines.

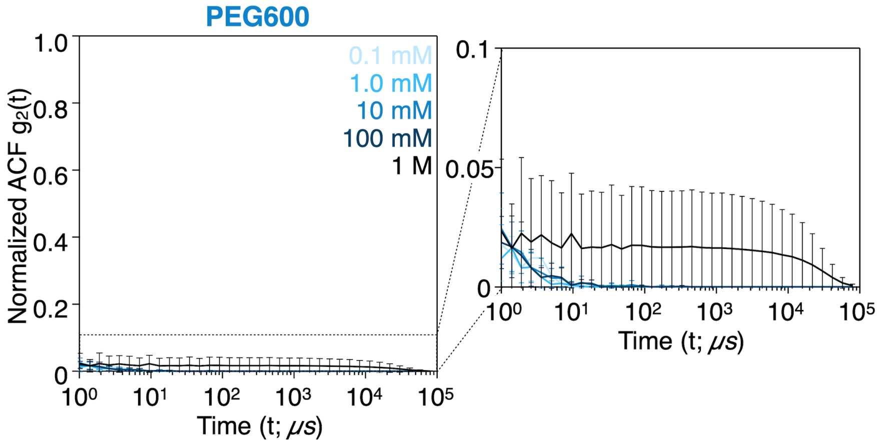

Figure S7. Normalized autocorrelation functions (ACFs) of PEG600 ([PEG600] = 0.1, 1.0, 10, 100 mM, 1 M) and its magnified graphs in water at 20 °C. Error bars represent the standard deviations of three independent experiments (n = 3).

8. Atomic Force Microscopy

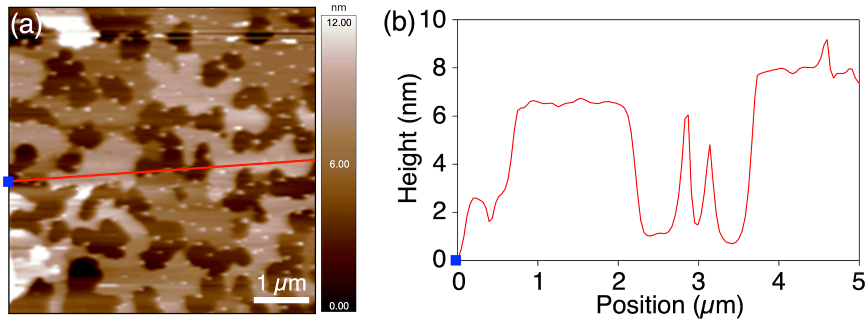

Figure S8. (a) AFM image of $^{p\text{-terph}}$OEG (0.1 mM) and (b) its height profiles along the cross-section indicated by the red line.
